# Individual differences in face identity processing

**DOI:** 10.1186/s41235-018-0112-9

**Published:** 2018-06-27

**Authors:** Jennifer M. McCaffery, David J. Robertson, Andrew W. Young, A. Mike Burton

**Affiliations:** 1 School of Psychology, University of Aberdeen, Aberdeen, UK; 2 Department of Psychology, University of York, York, YO10 5DD UK; 3 School of Psychological Sciences and Health, University of Strathclyde, Glasgow, UK

**Keywords:** Face recognition, Face perception, Unfamiliar faces, Familiar faces, Individual differences

## Abstract

We investigated the relationships between individual differences in different aspects of face-identity processing, using the Glasgow Face Matching Test (GFMT) as a measure of unfamiliar face perception, the Cambridge Face Memory Test (CFMT) as a measure of new face learning, and the Before They Were Famous task (BTWF) as a measure of familiar face recognition. These measures were integrated into two separate studies examining the relationship between face processing and other tasks. For Study 1 we gathered participants’ subjective ratings of their own face perception abilities. In Study 2 we used additional measures of perceptual and cognitive abilities, and personality factors to place individual differences in a broader context.

Performance was significantly correlated across the three face-identity tasks in both studies, suggesting some degree of commonality of underlying mechanisms. For Study 1 the participants’ self-ratings correlated poorly with performance, reaching significance only for judgements of familiar face recognition. In Study 2 there were few associations between face tasks and other measures, with task-level influences seeming to account for the small number of associations present. In general, face tasks correlated with each other, but did not show an overall relation with other perceptual, cognitive or personality tests. Our findings are consistent with the existence of a general face-perception factor, able to account for around 25% of the variance in scores. However, other relatively task-specific influences are also clearly operating.

**Electronic supplementary material:**

The online version of this article (10.1186/s41235-018-0112-9) contains supplementary material, which is available to authorized users.

## Significance statement

Perception and recognition of face identity is critical in many real-life contexts, including the identifications made by eye-witnesses and the inspection of passports or identity cards. The abilities involved in such tasks demand at least three things; ability to perceive identity in unfamiliar faces, ability to learn new faces, and ability to recognise familiar faces. Previous studies have shown substantial individual differences in such abilities, but without investigating all of them at the same time. We therefore investigated the relationships between individual differences in the performance of tasks that assess these different aspects of face-identity processing, using the Glasgow Face Matching Test (GFMT) as a measure of unfamiliar face perception, the Cambridge Face Memory Test (CFMT) as a measure of new face learning, and the Before They Were Famous task (BTWF) as a measure of familiar face recognition. These face-identity processing measures were integrated into two separate studies that investigated how they were themselves related to other factors. For Study 1 we devised a questionnaire-based measure of subjective performance in face perception and recognition. In Study 2 we used additional measures of perceptual and cognitive abilities, and personality factors, to place individual differences across our principal measures in a broader context. Our findings were consistent with the existence of a previously hypothesised general face-perception factor, but also showed that other influences are clearly operating, highlighting the potential for different aspects of face-perception abilities to associate with more general tasks in quite specific and differentiated ways.

## Background

The ability to perceive and recognise face identity is critical to real-life tasks ranging from eye-witness identification to passport control. Previous studies have consistently shown that such tasks reveal a wide distribution of ability in the normal population, and that training and experience do not much alter this (Burton, White, & McNeill, 2010; Dowsett & Burton, 2015; White, Kemp, Jenkins, & Burton, 2014; White, Kemp, Jenkins, Matheson, & Burton, 2014). The origins and nature of these individual differences have, therefore, become an issue with both theoretical and practical significance.

In terms of underlying theory, a key question in understanding face-identity recognition concerns the extent to which it can be considered a unitary process. Ways in which face recognition has already been considered non-unitary involve differences between perceiving and remembering individual face identity, and between the processing of familiar and unfamiliar faces. Since the theoretical paper by Bruce and Young (1986), both distinctions have been incorporated into widely discussed experimental paradigms, standardised tests, and cognitive models.

Much of this previous work was focussed on a case study approach based on examples from the extremes of ability, such as impairments following brain injury (Barton, 2008; Barton & Corrow, 2016; Young, 2011). In such studies, differences between the processing of the identities of familiar and unfamiliar faces are evident from double dissociations between neuropsychological impairments affecting unfamiliar face matching (e.g. in the Benton Test of Facial Recognition: Benton, Hamsher, Varney, & Spreen, 1983) and impairments of familiar face recognition (Benton, 1980; Malone, Morris, Kay, & Levin, 1982; Young, Flude, Hay, & Ellis, 1993; Young, Newcombe, de Haan, Small, & Hay, 1993). Likewise, the importance of new face learning is evident from neuropsychological cases where pre-morbidly familiar faces can be recognised but faces that have only been encountered since the brain injury go unrecognised (Hanley, Pearson, & Young, 1990; Hanley, Young, & Pearson, 1991; Ross, 1980).

While this tactic of studying dissociations between acquired deficits has been important and influential, it was often derived from an implicit assumption that there would be little variability in performance across the normal population. However, more recently it has become clear that there is actually quite a wide distribution of normal ability; especially for tasks involving unfamiliar faces. Hence, an individual-differences approach can be informative, in which one asks whether or not two putatively different types of face-identity recognition ability are associated with each other.

An individual-differences approach is now facilitated by the development of standardised tests. For example, the Glasgow Face Matching Test (GFMT: Burton et al., 2010), the Benton Test of Facial Recognition (Benton et al., 1983), and the Cambridge Face Perception Test (Duchaine, Germine, & Nakayama, 2007) have all been used to investigate the perception of unfamiliar face identity without any face-memory component, because they involve matching the identities of simultaneously presented images of unfamiliar faces. In contrast, the Cambridge Face Memory Test (CFMT: Duchaine & Nakayama, 2006) and the Warrington Recognition Memory Test (Warrington, 1984) look at the ability to recognise previously unfamiliar faces that have been learnt during the testing session. From these available tests we selected the widely used GFMT and CFMT to investigate potential differences between perceiving (GFMT) and remembering (CFMT) the identities of previously unfamiliar faces.

A recent study by Verhallen et al. (2017) found significant correlations between performance on the GFMT, CFMT, and two other measures of unfamiliar face perception. Similarly, Bowles et al. (2009) found a correlation between CFMT and the Cambridge Face Perception Test, and Davis, Lander, Evans, and Jansari (2016) reported a correlation between performance on the GFMT and an immediate memory test developed from the matching arrays used by Bruce et al. (1999). Verhallen et al. (2017) noted that this pattern of findings fits the idea of a general face perception factor, *f*, that might be considered analogous to Spearman's (1927) *g* in studies of measures of intelligence. At the same time, however, Verhallen noted that *f* should not be reified from a pattern of correlations alone, and that it can in any case account for only a proportion of the variance across face tests, with much unshared variance that still needs to be explained. A similar overall conclusion can be drawn from a previous study by Wilhelm et al. (2010), who used a different set of tasks to demonstrate an inter-relationship between measures of face processing that was largely distinct from other cognitive abilities.

Our aim here was to pursue a similar logic to Verhallen et al. (2017) while broadening the range of measures to include familiar face recognition. In terms of the possibility of differences between familiar and unfamiliar face recognition, much less is known from an individual differences perspective. In general, familiar face recognition is so much better than unfamiliar face recognition that it can be difficult to see how to measure individual differences in familiar face recognition (Young & Burton, 2017). For instance, familiar faces are so easily recognised across a wide range of views that identification performance is virtually at ceiling and matching tasks with familiar faces seem almost trivially easy (Jenkins, White, Van Montfort, & Burton, 2011). Nonetheless, it remains the case that people do make sporadic errors in familiar face recognition both in everyday life and in laboratory tasks (Hay, Young, & Ellis, 1991; Young, Hay, & Ellis, 1985) and also that some individuals (including at least one of the current authors) subjectively report experiencing more difficulty than others. So it is plausible that it might be possible to measure individual differences in familiar face recognition if a sufficiently demanding task can be devised.

One potentially promising measure of familiar face recognition is the Before They Were Famous task (BTWF: Russell, Duchaine, & Nakayama, 2009), which asks participants to recognise the identities of faces they know well from images of the same people before they came to public prominence. Performance on the BTWF has been found to correlate with CFMT (Wilmer et al., 2010), but the BTWF was designed for use with American participants, and many famous faces are culture-specific. We therefore used a slightly adapted version of the BTWF with faces appropriate for participants in the UK to explore individual differences in familiar face recognition. In their previous study, Russell et al. (2009) noted a significant association between the BTWF and the CFMT. Likewise, Davis et al. (2016) reported a correlation between a face-memory test and a famous face recognition test, though their famous face-recognition test involved visually degraded celebrity images rather than before-fame photos.

We therefore used an individual-differences approach to face-identity recognition based around three principal measures; the GFMT as a measure of unfamiliar face-identity perception, the CFMT as a measure of new face learning, and the BTWF as a measure of familiar face recognition. To the extent that these tap common processes such as Verhallen et al.’s (2017) *f*, we would expect correlations between the different measures.

These three principal measures were integrated into two separate studies that looked at how they were themselves related to other factors. For Study 1 we devised a questionnaire-based measure of subjectively experienced problems in face-identity perception and recognition. With the growing focus on individual differences in face-identity recognition, researchers have also begun to examine the extent to which individuals are aware of their own expertise (for a review, see Palermo et al., 2017). Despite this growing interest, approaches have been inconsistent and the findings contradictory. For face-memory tests, while Rotshtein, Geng, Driver, and Dolan (2007) found no correlation between a single self-report question and recognition accuracy, whereas Palermo et al. (2017), Gray, Bird, and Cook (2017) and Livingston and Shah (2017) did find some correlation between overall scores on multi-item face-metacognition questionnaires and CFMT performance. For face-matching tasks Bindemann, Attard, and Johnston (2014) reported no correlation between self-report ratings and performance on an unfamiliar face-matching task, whereas Verhallen et al. (2017) reported a significant correlation between a single self-report question and GFMT accuracy. To try and reconcile these discrepant findings, our questionnaire included both a general question and task-specific questions chosen to relate directly to the GFMT, CFMT and BTWF measures. Moreover, in contrast to several previous studies, our questions explicitly distinguished between familiar and unfamiliar faces.

In Study 1 we used a small and relatively homogeneous student participant sample to explore whether correlations between different face tests will be reasonably robust. For Study 2 we used a larger, more diverse sample of participants and additional measures of face perception, visual perception, cognitive abilities and personality factors to place individual differences across our principal measures in a broader context. Again, previous results have been mixed, but offered hints of possible relationships. Performance with unfamiliar faces has been associated with object-matching ability (Megreya & Burton, 2006), with perceptual style or field dependence (Hoffman & Kagan, 1977; Messick & Damarin, 1964; Witkin & Goodenough, 1977), and with space perception (Burgess, Alderman, Evans, Emslie, & Wilson, 1998). Personality traits of extraversion and empathy have been reported as positively associated with unfamiliar face recognition performance, whereas anxiety and neuroticism have been negatively associated with both unfamiliar face recognition and unfamiliar face matching (Bate, Parris, Haslam, & Kay, 2010; Li et al., 2010; Megreya & Bindemann, 2013; Mueller, Bailis, & Goldstein, 1979; Nowicki, Winograd, & Millard, 1979).

In Study 1, then, we provide the first assessment of individual differences in identity processing across three widely established tests that measure different aspects of face-identity recognition; the GFMT for unfamiliar face matching, CFMT for face learning, and the BTWF for familiar face recognition. In addition, we assessed awareness of one’s own aptitude on these tasks using both general and task-specific questions that dissociated face familiarity. In Study 2, using a more comprehensive battery of perceptual, cognitive, and personality measures, we sought to assess the extent to which aptitude with faces is domain-specific or correlates with other forms of individual difference.

## Study 1

In this study we measured the extent to which accuracy on tests of unfamiliar face matching (GFMT), memory for newly learnt faces (CFMT), and familiar face recognition (BTWF) were associated in a student population. In addition, we created a questionnaire-based subjective measure to determine the extent to which participants’ estimates of their ability with faces was correlated with the more objective measures.

## Methods

### Participants

Forty participants aged between 17 and 30 years (*M* = 21 years, standard deviation (SD) = 4, nine male) were recruited from the University of Aberdeen. All participants had normal or corrected-to-normal vision. The University of Aberdeen, School of Psychology Ethics Committee, approved the study. All participants provided written informed consent; half of the sample completed the study as part of a course requirement, and the remaining half received a small monetary payment.

### Stimuli and tasks

#### The Glasgow Face Matching Test (GFMT – short form)

The short version of the GFMT (Burton et al., 2010) is an unfamiliar face-matching task consisting of 40 pairs of simultaneously presented unfamiliar faces, half of which are same identity face pairs and half of which are different identity face pairs. Each face image is front facing in pose, neutral in expression, shown in colour and standardised to a width of 151 pixels. In order to ensure that the GFMT would provide a non-trivial matching task, the photos within each pair were taken a few minutes apart using different cameras (for more details see Burton et al., 2010). Participants were asked ‘Are the images of the same individual?’ and were required to indicate, via a keypress, whether each pair of face photos depicted the same person (press ‘1’) or two different people (press ‘2’). The task was self-paced, and typically took about 5 min to complete.

#### The Cambridge Face Memory Test (CFMT)

The CFMT (Duchaine & Nakayama, 2006) is a 72-item recognition-memory task, involving learning the identities of previously unfamiliar faces, which is split into three sections. In section 1, participants are told to learn a target face shown in three orientations (left facing, forward facing, right facing). Participants are then presented with a three-alternative forced choice task in which they have to pick out the identical face image. This process is repeated for each of six target faces. In section 2 the three-AFC test is retained, with participants now having to identify novel instances of each target face. Section 3 is identical to section 2, with the exception that the test images have had visual noise added to them in order to make the task more challenging. Presentation of the stimuli for memorising was timed; presentation of the target stimuli was not. There was no time limit for responses and the task typically took about 15 min to complete.

#### The Before They Were Famous task (BTWF)

The BTWF is a measure of familiar face recognition that we adapted from Russell et al. (2009). Our adapted version of the task consisted of a recognition test involving 40 photos of celebrities known in the UK that were taken before they became famous (i.e. photos taken when they were children or adolescents). To ensure participants were familiar with the celebrities we added a familiarity test involving 40 photos of the same celebrities as adults (i.e. photos taken during their period of fame). Each photo was cropped to capture the celebrity’s face and all of the images were standardised to a height of 400 pixels and were shown in greyscale or colour, depending on the age of the photograph. Participants were presented with each of the before-fame celebrity photos and, in line with the instructions used in Russell et al. (2009), were required to indicate the celebrity’s name (e.g. Daniel Craig) or, alternatively, provide a unique identifying description (e.g. the actor who plays the current James Bond). The same procedure was then used to test recognition of the same celebrities with photos dating from their period of fame.

In contrast to the BTWF as used by Russell et al. (2009), the present task was adapted to include a familiarity post-test in which participants were also presented with adult (when-famous) photos of each of the celebrities. This was done because in the Russell et al. (2009) version of the test, a high score might simply indicate that the participant knew a wider selection of the chosen celebrities, rather than providing a direct indication of familiar face-recognition ability per se. Therefore, in the present version of the task, familiar face-recognition performance was calculated as the percentage of celebrities a participant recognised from the when-famous photographs that were recognised by the same participant from the photographs of them before they became famous (see Bindemann et al., 2014). The task was self-paced and typically took about 15 minutes to complete.

#### The Face Recognition Ability Questionnaire

The questionnaire used to test participants’ estimates of their face-recognition ability consisted of six questions drawn from findings in the research literature on face recognition. Question 1 asked participants for an overall estimate of their ability, question 2 asked specifically about unfamiliar face matching (cf. GFMT), question 3 asked about familiar face recognition (cf. BTWF), questions 4–5 asked for estimates of face learning (cf. CFMT), and question 6 probed insight into recent observations that seeing multiple different views of a face promotes the learning of face identity (see Andrews, Jenkins, Cursiter, & Burton, 2015).

For questions 1–2 and 4–6, participants were asked to rate their ability on a 9-point Likert scale ranging from ‘below average’ (1) to ‘above average’ (9). For question 3, the number of faces given by participants was used as the measure.

- *Question 1*: Some people are better than average at face recognition. These people are known as super-recognisers. How would you rate your face-recognition ability in general (that is the ability to recognise new instances of people you are familiar with, to recognise new instances of a person that you have seen just once, or with whom you are relatively unfamiliar)?
- Question 2*:* Imagine that you were presented with pairs of unfamiliar faces, some of those pairs of faces would show two different photos of the same face, while others would should the faces of two different people who look very similar. How likely is it that you would correctly detect whether the face pairs depicted the same person, or different people?
- *Question 3*: Some television shows challenge people to recognise current celebrities by showing photos of them as children (i.e. before they were famous). If you were provided with 40 childhood photos of current celebrities that you know, how many of the celebrities do you think you would be able to recognise from their childhood photos?
- *Question 4*: If I were to show you one picture of a person you are unfamiliar with, how likely is it that you would be able to recognise the same photo of that person at a later date?
- *Question 5*: If I were to show you one photo of a person you are unfamiliar with, how likely is it that you recognise a different photo of that person at a later date?
- *Question 6*: If I were to show you several pictures of a person that you are unfamiliar with, how likely is it that you would be able to recognise a different photo of that person at a later date?

### Procedure

Participants first completed the Face Recognition Ability Questionnaire, followed by the three face-identity processing tests. The questionnaire was given first so that responses would not be affected by participants’ opinions concerning their subsequent performance of the face tests themselves. The order of presentation of the face-identity tests was then counterbalanced across participants. The questionnaire was administered on paper and the face-processing tasks were administered using E-Prime 2.0 and a JavaScript Applet.

## Results

Five participants were removed from all analyses either as a result of software failure during data collection (three participants), or because they scored more than 2.5 SDs below the mean on the BTWF familiarity check (two participants). In addition, a sixth participant failed to complete the Face Recognition Ability Questionnaire, leaving 34 participants for the analyses involving the questionnaire data.

For the GFMT, mean unfamiliar face matching accuracy was 82% (SD = 11%). Mean CFMT face memory accuracy across all items in the task was 74% (SD = 14%). These levels of performance are in line with published norms (GFMT, Burton et al., 2010; CFMT, Russell et al., 2009). For the BTWF, the familiarity check established that the mean number of known celebrities was 70% (SD = 21%). Mean familiar face recognition accuracy for the known celebrities from the images before they were famous was 27% (SD = 13%). This recognition rate is line with previously published work by Bindemann et al. (2014), and the range of scores is consistent with those reported by Russell et al. (2009).

**Fig. 1:**
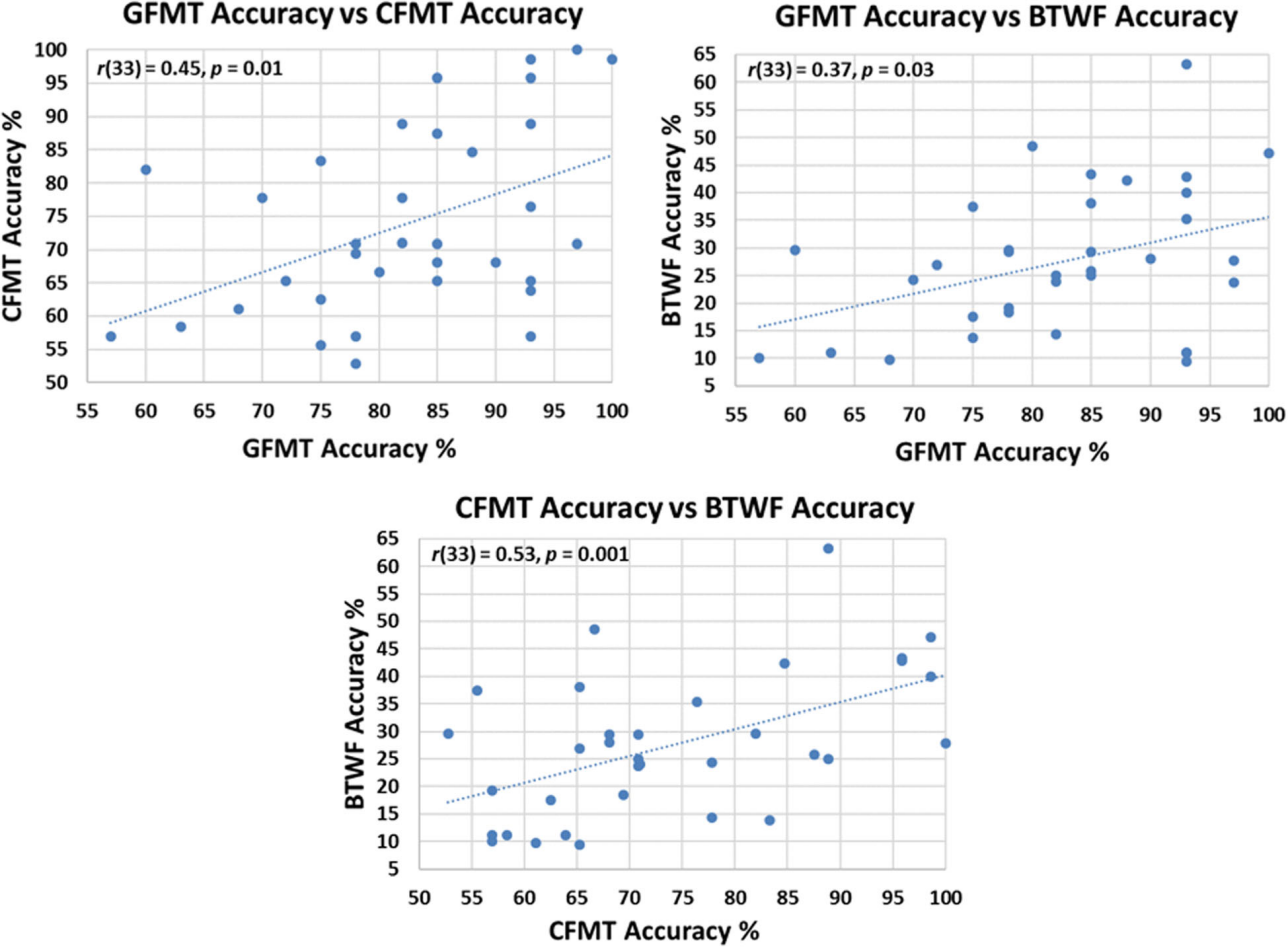
Scatterplots showing the correlations between performance on the Glasgow Face Matching Test (GFMT) and the Cambridge Face Memory Test (CFMT) (upper left), the GFMT and the Before They Were Famous (BTWF) task (upper right), and the CFMT and BTWF (lower) in Study 1

As our principal aim was to explore potential correlations between different measures, we were more concerned to avoid type 2 than type 1 errors, and therefore begin by reporting uncorrected statistics. As a check on the reliability of these, however, we also used the Benjamini-Hochberg procedure with a false discovery rate of 0.2 to correct for multiple comparisons, and we also report confidence intervals. Fig. 1 provides scatterplots showing the correlation between performance on the three face-identity processing tasks. Significant positive Pearson correlations were found between the GFMT and CFMT (*r*(33) = .45, uncorrected *p* = .006, 95% CI [0.14, 0.68]), GFMT and BTWF (*r*(33) = .37, uncorrected *p* = .027, 95% CI [0.04, 0.62]), and CFMT and BTWF (*r*(33) = .53, uncorrected *p* = .001, 95% CI [0.24, 0.73]), showing some consistency in performance. All of these correlations remained significant with the Benjamini-Hochberg correction.

Table 1 shows correlations between the three face-identity processing tasks (GFMT, CFMT, and BTWF) and the six items from our questionnaire (see Additional file 1 for raw data). Significant correlations were only found for question 3. Interestingly, this question asked about familiar face recognition and it correlated with our performance measures for familiar face recognition (BTWF, *r*(32) = .42, uncorrected *p* < .05, 95% CI [0.09, 0.66]) and face learning (CFMT, *r*(32) = .48, uncorrected *p* < .01, 95% CI [0.17, 0.70]), with both correlations remaining significant after Benjamini-Hochberg correction, suggesting that people may have at least some insight into their level of ability with familiar or learnt faces. In general, though, the most striking aspect is of these results is how poorly predictive people’s subjective view of their abilities are with unfamiliar faces.

**Table 1:** Correlations between Questionnaire items (see ‘Methods’ for exact wording of each question) and face identity measures (Glasgow Face Matching Test, GFMT; Cambridge Face Memory Test, CFMT; Before They Were Famous task, BTWF) from Study 1. Uncorrected significant correlations are shown in bold: ^a^*p* < .05, ^b^*p* < .01; ^c^Significant correlations following Benjamini-Hochberg correction; 95% CI shown in brackets

	GFMT	CFMT	BTWF
Question 1: Participant’s overall estimate of their ability	.30 [− 0.04, 0.57]	.25 [− 0.09, 0.54]	.14 [− 0.21, 0.45]
Question 2: Participant’s estimate of their ability to match unfamiliar faces (cf. GFMT)	.01 [− 0.32, 0.34]	−.13 [− 0.44, 0.21]	−.17 [− 0.47, 0.18]
Question 3: Participant’s estimate of their ability to recognise familiar faces (cf. BTWF)	.16 [− 0.19, 0.47]	**.48** ^**bc**^ [0.17, 0.70]	**.42** ^**ac**^ [0.09, 0.66]
Question 4: Participant’s estimate of their ability to remember a photo of an unfamiliar face (cf. CFMT)	.10 [− 0.24, 0.42]	.18 [− 0.17, 0.48]	.14 [− 0.21, 0.45]
Question 5: Participant’s estimate of their ability to remember an unfamiliar face and recognise it from a different photo (cf. CFMT)	−.10 [− 0.42, 0.24]	.24 [− 0.11, 0.53]	.25 [− 0.09, 0.54]
Question 6: Participant’s insight into whether seeing multiple different views of a face will promote the learning of face identity	−.16 [− 0.47, 0.19]	.16 [− 0.19, 0.47]	.20 [− 0.15, 0.50]

## Study 2

In Study 1 we found that performance across unfamiliar face matching (GFMT), unfamiliar face learning (CFMT), and familiar face recognition (BTWF) were significantly correlated in a student sample.

Study 2 sought to replicate and extend the findings of Study 1 with a larger participant sample with a wider demographic and educational background, and at the same time to investigate the relationship to other measures.

We added two face-perception tasks that did not require recognition of individual identity (Face-detection, and Mooney faces), allowing us to explore their relationship to the identity-related face tasks (GFMT, CFMT, and BTWF). These additional face-perception measures were chosen because they tap theoretically important aspects of face perception without specifically requiring processing of face identity per se. Face detection was used because, since Ellis (1983), some theories have assumed that detecting faces in cluttered visual scenes forms an essential ‘entry point for all other tasks with faces’ (Bindemann & Lewis, 2013, p.1144). The Mooney face task is of interest because, even though it does not require perception of unique identity, it has been linked to genetic differences in ability (Verhallen et al., 2014) that may also be tapped by the CFMT (Shakeshaft & Plomin, 2015). Moreover, the Mooney face task has been found to correlate with CFMT in a previous study (Verhallen et al., 2017).

A battery of more general tasks was also used to identify the extent to which different face-processing tasks might associate with other types of task in relatively specific ways, using a similar logic to Wilhelm et al. (2010). These tasks included measures of visual space perception, visual object perception, executive function, and personality. As noted in the ‘Background’ section, although previous results have been mixed, these are all areas that have offered hints of possible relationships.

## Methods

### Participants

Participants were 103 members of the public from the Qualtrics panel and four students at the University of Aberdeen (51 male) who all reported normal or corrected to normal vision (mean age = 53 years, SD = 15, range = 21–82). The University of Aberdeen, School of Psychology Ethics Committee, approved the study. All participants provided informed consent and completed the study in their own time on their own computers via the Qualtrics online survey system. Participants received a small monetary payment.

### Stimuli and tasks

All participants completed five face-processing measures. Three of these, the GFMT, CFMT, and BTWF were as used in Study 1. The additional face-processing measures involved face detection and perception of Mooney faces.

#### Face-detection task

The Face-detection task measured participants’ ability to detect hidden faces in artworks. For example, Salvador Dali’s painting ‘*Man/couple with sleeping dogs’* (1948) comprises a central figure with other faces embedded in the scene. Participants were shown Dali’s painting as a practice image and encouraged to find the faces. Immediately following this, they were shown two test images for 1 min each. These were the artworks ‘*The forest has eyes*’ (1984) by Bev Doolittle, and ‘*Party*’ (2010) by Y&R, Dubai, for LG Viewty. Copyright restrictions prevent us reproducing these here, but all three works can easily be found through Internet search. Performance was scored as the total number of faces found across the two test trials.

#### Mooney face task

**Fig. 2:**
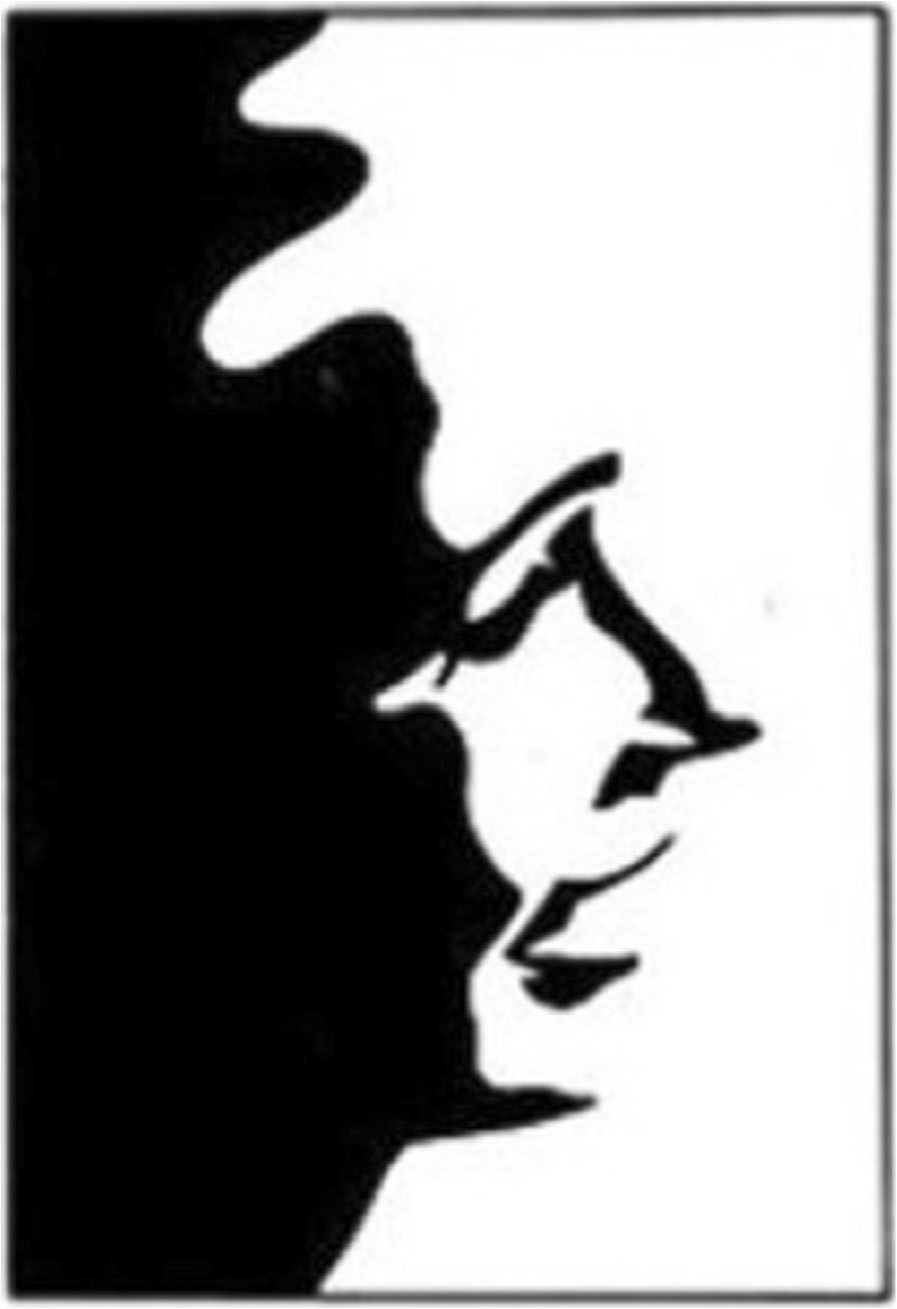
Examples of a Mooney face

This task (Mooney, 1957) measures perceptual closure using high-contrast face images consisting of exclusively dark or light regions. The test comprises 40 high-contrast images created from 20 male faces and 20 female faces; an example is shown in Fig. 2. Participants are required to identify whether each image shows a male or a female face. The task was self-paced and typically took about 5 min to complete.

#### Additional measures

All participants also completed 10 additional measures of visual perception, cognition, and personality, as described below.

A. Visual space perception

1. *Visual Object and Space Perception (VOSP Battery) Position Discrimination Task* (Warrington & James, 1991)*.* This is a subtask of the VOSP Battery measuring visual space perception. Participants view one of 20 pairs of squares, each containing a single black dot, and are required to identify in which of the squares the spot is more precisely central. The task was self-paced and typically took about 5 min to complete
2. *Birmingham Object Recognition Battery (BORB) Position of Gap Match* (Riddoch & Humphreys, 1993). This is a visual space perception task measuring location discrimination. A self-paced task in which participants view 40 pairs of circles, each with a small gap, and identify whether the gaps in the circles are in exactly the ‘Same’ position or slightly ‘Different’ positions. The task was self-paced and typically took about 5 min to complete

B. Visual object perception

3) *Letter Detection Task.* This was an in-house developed task in which participants were required to read a passage about ‘France’ and count the number of letter ‘f’s in the text. The aim was to create a parallel to the Face-detection Task that did not involve faces. The text passage was 300 words long and contained 50 ‘f’s. Answers were scored in terms of the total number of letters correct. Participants were given 1 min to complete the task
4) *Navon Global Task* (based on Navon, 1977)*.* The Navon Global Task consisted of 24 presentations of a display involving either a large letter ‘H’ or ‘S’ which was itself made up of either small letter ‘H’s or ‘S’s. Each display was presented for 5 s. Participants were required to give the identity of the large letters by using the mouse to click on ‘H’ or ‘S’. Participants’ responses were recorded for accuracy and time. The task typically took about 5 min
5) *Navon Local Task* (based on Navon, 1977)*.* This is a local processing task, with each letter appearing on screen for 5 s. The Navon Local Task consisted of 24 presentations of a large letter ‘H’ or ‘S’ made up of either small letter ‘H’s or ‘S’s. Participants were required to identify the identity of the small letters by using the mouse to click on ‘H’ or ‘S’. Participants’ responses were recorded for accuracy and time and the task typically took about 5 min
6) *Matching Familiar Figures Test* (Kagan, 1965*).* This task has been used to measure the cognitive style of impulsivity versus reflexivity. However, following Megreya and Burton (2006) we used the stimuli as an object-matching task. The Matching Familiar Figures Test involves 40 line drawings of common objects in which a target object drawing is depicted together with an array of six minor variants. Participants are required to identify the variant that precisely matches the target. The task was self-paced and typically took about 10 min to complete
7) *Visual Object and Space Perception (VOSP Battery) Silhouettes Task* (Warrington & James, 1991). This is a subtask of the VOSP Battery measuring visual object recognition under demanding conditions. Participants are required to identify common objects seen in silhouette from unusual perspectives. The stimuli consist of 15 animals and 15 objects. The task was self-paced and typically took about 10 min to complete

C. Executive function

8) *Behavioural Assessment of the Dysexecutive Syndrome (BADS) Card Sorting Task* (Wilson, Alderman, Burgess, Emslie, & Evans, 1997)*.* This is a rule-shifting task measuring cognitive flexibility and inhibition. Participants view 20 images of playing cards individually and in part 1 have to answer ‘Yes’ or ‘No’ to the question ‘Is the card red?’ In part 2 of the task the rule is changed and participants see the cards again but have to adapt their responses and inhibit their original response to answer ‘Yes’ or ‘No’ to the question ‘Is the card the same colour as the previous card?’ This was given as a self-paced task that typically took about 5 min to complete

D. Personality measures

9) *Big Five Inventory (BFI)* (John, Donahue, & Kentle, 1991)*.* This is a 44-item questionnaire measuring five widely-used personality factors; Openness, Conscientiousness, Extraversion, Agreeableness, and Neuroticism. Each question consists of a short phrase such as ‘I am always prepared’. Participants must state how accurately this reflects their experience by selecting the most appropriate response from a 5-point Likert scale ranging from ‘disagree strongly’ to ‘agree strongly’. The task was self-paced and typically took about 5 min to complete
10) *Interpersonal Reactivity Index (IRI)* (Davis, 1980)*.* This is a 28-item questionnaire measuring four independent empathy factors; Empathic Concern, Personal Distress, Perspective Taking, and Fantasy. Each question consists of a short phrase such as ‘After seeing a play or a movie, I have felt as though I am one of the characters’. Participants must state how accurately this reflects their experience by selecting the most appropriate response from a 5-point Likert scale ranging from ‘does not describe me well’ to ‘describes me very well’. The task was self-paced and typically took about 5 min to complete

### Procedure

This study was conducted on-line using the Qualtrics survey system. The output code and data analysis were generated using Version 2015 of the Qualtrics Research Suite software; see http://www.qualtrics.com. Participants were tested individually and completed all of the measures. The face-identity processing tasks were presented first in randomised order to avoid any potential confounds from the other tasks. Participants then completed the remaining tasks in randomised order.

## Results

Summary statistics for data from the measures used in Study 2 are presented in Table 2 (see Additional file 1 for raw data). Scores from the relatively objective measures (face processing, visual space perception, visual object perception, and executive function) have been converted to percentage correct, to facilitate comparison.

**Table 2:** Summary statistics for data from all measures used in Study 2. Face-processing tasks included measures of face-identity recognition (Glasgow Face Matching Test GFMT, Cambridge Face Memory Test CFMT, Before They Were Famous task BTWF) and face perception (Face-detection, Mooney faces)

Measure	Mean	Standard deviation	Range	Minimum	Maximum
Face processing:					
GFMT (%)	80.75	10.87	52.50	47.50	100.00
CFMT (%)	69.85	15.06	66.67	31.94	98.61
BTWF (%)	15.58	12.40	55.26	0.00	55.26
Face-detection (%)	56.70	14.79	60.00	26.67	86.67
Mooney faces (%)	84.38	12.68	100.00	0.00	100.00
Visual space perception:					
VOSP position discrimination (%)	97.38	4.42	20.00	80.00	100.00
BORB gap position (%)	85.70	13.57	60.00	40.00	100.00
Visual object perception:					
Letter detection (%)	48.88	18.63	100	0.00	100.00
Navon global (%)	92.48	16.50	50.00	50.00	100.00
Navon local (%)	95.44	13.18	50.00	50.00	100.00
Matching Familiar Figures (%)	77.31	12.24	57.50	40.00	97.50
VOSP Silhouettes (%)	73.02	17.44	100.00	0.00	100.00
Executive function:					
BADS Card Sorting (%)	97.71	4.87	30.00	70.00	100.00
Personality:					
Big 5 Extraversion	24.82	6.31	29.00	9.00	38.00
Big 5 Agreeableness	32.85	5.76	27.00	18.00	45.00
Big 5 Conscientiousness	34.06	5.37	22.00	23.00	45.00
Big 5 Neuroticism	22.94	6.19	31.00	8.00	39.00
Big 5 Openness	35.09	6.11	33.00	17.00	50.00
IRI Perspective Taking	16.92	4.26	21.00	7.00	28.00
IRI Fantasy Scale	13.64	4.57	21.00	5.00	26.00
IRI Empathic Concern	18.51	4.41	20.00	8.00	28.00
IRI Personal Distress	11.32	4.20	22.00	0.00	22.00

**Fig. 3:**
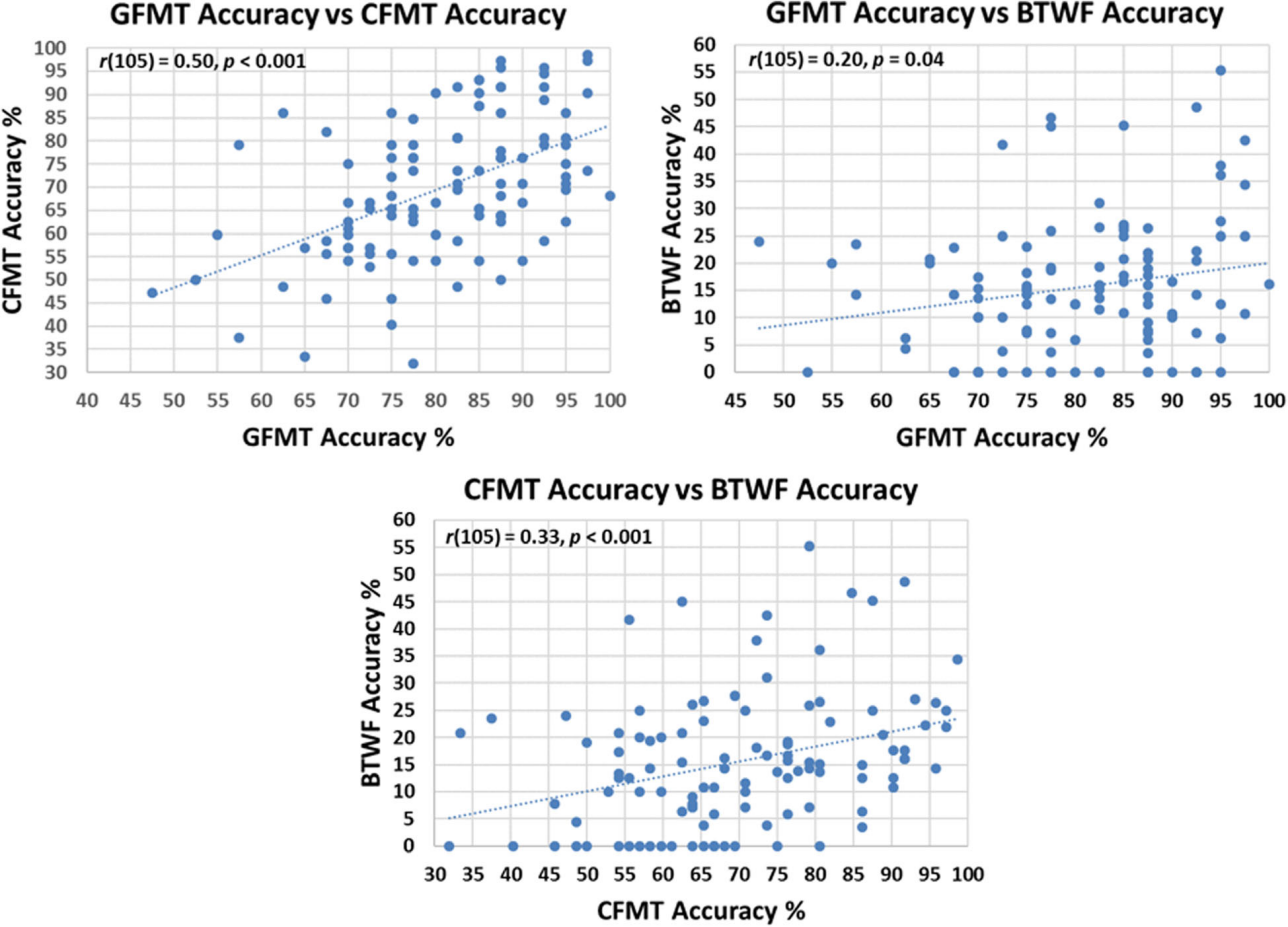
Scatterplots showing the correlations between performance on the three core face tests in Study 2

We began by examining the correlations between performance on the three face-identity tasks that had also formed the core of Study 1(GFMT, CFMT, and BTWF). Scatterplots are shown in Fig. 3. As for Study 1, significant positive correlations were found between the GFMT and CFMT (*r*(105) = .50, uncorrected *p* < .001, 95% CI [0.35, 0.63]), GFMT and BTWF (*r*(105) = .20, uncorrected *p* = .039, 95% CI [0.01, 0.37]), and CFMT and BTWF (*r*(105) = .33, uncorrected *p* < .001, 95% CI [0.15, 0.49]). However, the correlations with the BFTW task were somewhat lower than for Study 1, and only the correlations between GFMT and CFMT and between CFMT and BTWF remained significant following the Benjamini-Hochberg correction. Figure 3 shows clearly that correlations involving BTWF were likely reduced because a number of participants in this more heterogeneous sample were at floor on the adapted BTWF; 20% of participants failed to recognise any of the celebrities as children, despite recognising a mean of 59% (SD = 24%) of the celebrities as adults.

Table 3 shows the relationships between performance accuracy across the three face-identity-processing tasks (GFMT, CFMT, BTWF) and the additional face-perception tasks included in Study 2. Significant but modest correlations were noted between the Mooney face task and CFMT (*r*(105) = .22, uncorrected *p* = .022, 95% CI [0.03, 0.39]), and between Face-detection and Mooney faces (*r*(105) = .20, uncorrected *p* < .01, 95% CI [0.11, 0.45]). Both correlations remained significant with the Benjamini-Hochberg correction.

**Table 3:** Correlations between performance on the additional face-processing tasks (Face-detection and Mooney faces) and face-identity tasks (Glasgow Face Matching Test (GFMT), Cambridge Face Memory Test (CFMT), Before They Were Famous task (BTWF)) used in Study 2. Uncorrected significant correlations are shown in bold: ^a^*p* < .05, ^b^*p* < .01; ^c^Significant correlations following Benjamini-Hochberg correction; 95% CI shown in brackets

	Face-detection	Mooney faces
GFMT	.10 [− 0.09, 0.28]	.10 [− 0.09, 0.28]
CFMT	.13 [− 0.06, 0.31]	**.22** ^**ac**^ [0.03, 0.39]
BTWF	**.**13 [− 0.07, 0.31]	.17 [− 0.07, 0.31]
Face-detection		**.29** ^**bc**^ [0.11, 0.45]

Table 4 shows the correlations between performance on the five face-processing tasks (GFMT, CFMT, BTWF, Face-detection, and Mooney faces) and the additional perceptual, cognitive and personality measures. Differences and similarities found here may provide insight into underlying relationships.

**Table 4:** Correlations between performance on the face-processing tasks (Face-detection and Mooney faces) and face-identity tasks (Glasgow Face Matching Test GFMT, Cambridge Face Memory Test (CFMT), Before They Were Famous task (BTWF)) and other perceptual, cognitive and personality measures in Study 2. Uncorrected significant correlations are shown in bold: ^a^*p* < .05, ^b^*p* < .01, ^c^*p* < .001; ^d^Significant correlations following Benjamini-Hochberg correction; 95% CI shown in brackets

Measure	GFMT	CFMT	BTWF	Face-detection	Mooney faces
Visual space perception:					
VOSP position Discrimination	.10 [−0.09, 0.28]	.14 [−0.05, 0.32]	.06 [−0.13, 0.24]	−.03 [−0.22, 0.16]	.09 [− 0.1, 0.27]
BORB gap position	.13 [− 0.06, 0.31]	−.05 [− 0.23, 0.14]	.06 [− 0.13, 0.25]	.01 [− 0.18, 0.2]	**.33** ^**cd**^ [0.15, 0.49]
Visual object perception:					
Letter detection	.14 [− 0.05, 0.32]	.07 [− 0.12, 0.26]	.08 [− 0.11, 0.26]	**.27** ^**bd**^ [0.09, 0.44]	.06 [− 0.13, 0.24]
Navon global	−.01 [− 0.19, 0.18]	−.10 [− 0.28, 0.1]	.09 [− 0.1, 0.27]	.16 [− 0.03, 0.34]	−.00 [− 0.19, 0.19]
Navon local	**.27** ^**bd**^ [0.09, 0.44]	.03 [− 0.16, 0.22]	.03 [− 0.16, 0.22]	.16 [− 0.03, 0.34]	−.01 [− 0.2, 0.18]
Matching Familiar Figures	**.37** ^**cd**^ [0.19, 0.52]	.02 [− 0.16, 0.21]	−.00 [− 0.19, 0.19]	.06 [− 0.13, 0.25]	.08 [− 0.11, 0.26]
VOSP Silhouettes	.14 [− 0.05, 0.32]	**.29** ^**bd**^ [0.11, 0.45]	**.29** ^**bd**^ [0.10, 0.45]	.12 [− 0.07, 0.30]	**.50** ^**cd**^ [0.34, 0.63]
Executive function:					
BADS Card Sorting	**.20** ^**a**^ [0.01, 0.38]	.12 [− 0.08, 0.30]	.14 [− 0.06, 0.32]	−.13 [− 0.31, 0.06]	.06 [− 0.13, 0.25]
Personality:					
Big 5 Extraversion	.03 [− 0.16, 0.22]	.00 [− 0.19, 0.19]	.04 [− 0.15, 0.22]	−.05 [− 0.15, 0.23]	.07 [−0.12, 0.26]
Big 5 Agreeableness	−.05 [−0.24, 0.14]	−.07 [− 0.25, 0.13]	.02 [− 0.17, 0.21]	−.14 [− 0.32, 0.05]	−.01 [− 0.19, 0.18]
Big 5 Conscientiousness	.17 [− 0.02, 0.35]	.08 [−0.11, 0.26]	−.03 [− 0.22, 0.16]	−.07 [− 0.25, 0.12]	−.05 [− 0.23, 0.15]
Big 5 Neuroticism	.02 [− 0.17, 0.21]	−.05 [− 0.23, 0.14]	−.04 [− 0.22, 0.15]	.02 [−0.17, 0.21]	−.01 [− 0.2, 0.18]
Big 5 Openness	.**23**^**ad**^ [0.04, 0.40]	.11 [−0.08, 0.30]	.01 [− 0.1, 0.28]	.12 [− 0.07, 0.31]	.13 [− 0.06, 0.31]
IRI Perspective Taking	.17 [− 0.02, 0.35]	.08 [− 0.12, 0.26]	.13 [− 0.06, 0.31]	.03 [− 0.16, 0.22]	.03 [− 0.16, 0.22]
IRI Fantasy Scale	.16 [− 0.03, 0.34]	.07 [− 0.12, 0.26]	.15 [−0.04, 0.33]	.05 [− 0.14, 0.24]	−.05 [− 0.24, 0.14]
IRI Empathic Concern	**.20** ^**a**^ [0.01, 0.38]	.06 [− 0.13, 0.24]	.06 [− 0.13, 0.24]	−.06 [− 0.24, 0.14]	.10 [− 0.1, 0.28]
IRI Personal Distress	−.10 [− 0.28, 0.09]	.01 [− 0.19, 0.19]	−.06 [− 0.24, 0.13]	.03 [−0.16, 0.21]	−.02 [− 0.21, 0.17]

The most striking aspect of Table 4 is the apparent lack of clear associations between our face tasks and other variables. In particular, none of the non-facial tasks was correlated with all of the face-identity tasks. This pattern is strikingly different to the consistent intercorrelations between the face-identity tasks themselves (as shown in Figs. 1 and 3), suggesting that if there is a generic face factor (*f*) it represents something that is relatively distinct from other abilities.

Nonetheless, a few correlations did reach significance at both uncorrected and corrected levels. Some of these seem to reflect relatively isolated influences, such as the correlation between letter detection and face detection (*r*(105) = .27, uncorrected *p* < .01, 95% CI [0.09, 0.44]) and the correlation between BORB Gap Detection and Mooney faces (*r*(105) = .33, uncorrected *p* < .001, 95% CI [0.15, 0.49]), but two more general patterns also stand out.

First, inspection of the data in terms of the columns in Table 4 shows that unfamiliar face matching (the GFMT) correlates with other matching tasks that require careful local processing (Navon Local, *r*(105) = .27, uncorrected *p* = .004, 95% CI [0.09, 0.44]; Matching Familiar Figures, *r*(105) = .37, uncorrected *p* <. 001, 95% CI [0.19, 0.52]) and with our test of executive function (BADS Card Sorting, *r*(105) = .20,uncorrected *p* = .035, 95% CI [0.01, 0.38]), though the latter correlation was not significant after correction for multiple comparisons. This pattern is consistent with the idea that unfamiliar face matching is often done using feature processing strategies that can be bolstered by a degree of careful strategic control and systematic checking of individual features (Megreya & Burton, 2006).

Second, inspection of the data across rows in Table 4 shows that the measure of visual object recognition (VOSP Silhouettes) correlates with the measures of familiar face recognition (BTWF, *r*(105) = .29, uncorrected *p* = .003, 95% CI [0.10, 0.45]) and face learning (CFMT, *r*(105) = .29, uncorrected *p =* .002, 95% CI [0.11, 0.45]). It also correlates with the Mooney face task (*r*(105) = .50, uncorrected *p* < .001, 95% CI [0.34, 0.63]), but this is perhaps unsurprising as both tasks have a requirement that involves interpreting high-contrast images.

Although correlations between measures of face perception and personality have been reported in some previous studies (Bate et al., 2010; Li et al., 2010; Megreya & Bindemann, 2013; Mueller et al., 1979; Nowicki et al., 1979), Table 4 does not show strong relationships.

Response times were also available for some measures (Navon global, Navon local, Matching Familiar Figures, BADS Card Sorting), but these did not show significant correlations with any of the accuracy measures and hence are not discussed further here.

## Discussion

Our aims were to investigate the relationships between individual differences in the performance of three tasks that assessed different aspects of face-identity processing, and to investigate the relationships between these tasks and other measures of face perception and broader perceptual, cognitive, and personality measures.

Our three principal measures of face-identity processing involved the GFMT as a measure of unfamiliar face perception, the CFMT as a measure of face learning, and the BTWF as a measure of familiar face recognition. In both Study 1 and Study 2, performances were significantly correlated across these tasks, consistent with some degree of commonality. This is the first time that this has been shown across unfamiliar face matching, unfamiliar face learning, and familiar face recognition at the same time.

While our sample sizes were small for definitive correlation-based research (Schönbrodt & Perugini, 2013), the significant intercorrelations between the face measures across both studies nonetheless lend further support to Verhallen et al.’s (2017) concept of a general face-perception factor, *f*. In fact they extend the range of applicability of the idea since Verhallen et al. did not include a measure of familiar face recognition. That said, the intercorrelations we noted could at best account for around 25% of the variance in test scores, and this is consistent with Verhallen et al.’s (2017) finding that the upper limit on the proportion of variance explained in their study was 23%. So even though it may be appropriate to think in terms of a general face factor, there seem also to be other influences operating.

Our three principal measures of GFMT, CFMT, and BTWF were also integrated into separate studies that looked at how they were themselves related to other factors. For Study 1 we devised a questionnaire-based measure of subjective problems in face perception and recognition. Of the six different questions we devised, only the question concerning familiar face recognition (question 3, see Table 1) led to significant correlations with our measures of face recognition (BTWF) and face learning (CFMT). It seems that participants may at least have some insight into their familiar face-recognition abilities, while having little insight into their unfamiliar face recognition. This in itself is not an entirely new finding; Bindemann et al. (2014) also noted greater associations between perceived ability and accuracy by splitting predictions across unfamiliar and familiar face tasks. The pattern is consistent with the interesting theoretical idea that it is the importance of familiar face recognition to our everyday lives that leads many of us to fail to appreciate just how error-prone tasks like unfamiliar face matching can be (Ritchie et al., 2015; Young & Burton, 2017).

In Study 2 we used additional measures of face perception, visual perception, cognitive abilities, and personality factors to place individual differences across our principal measures in a broader context. While Studies 1 and 2 both found that face-identity processing tasks were significantly associated with each other, Study 2 nonetheless showed that they still seemed to involve some specific components. There were no general tasks or measures that were associated with all of the face-identity processing tasks, suggesting that any general face factor (*f*) may represent something that is relatively distinct from other abilities. However, we noted that the hypothesised *f* can at best explain around 25% of the variability in face-task scores. Consistent with this, there was a smattering of individual tasks and measures that did associate with specific face-processing tasks.

Of particular note, unfamiliar face matching (the GFMT) correlated with other matching tasks that require careful local processing (Navon Local, Matching Familiar Figures) or executive function (BADS Card Sorting). We interpret this pattern as consistent with the idea that unfamiliar face matching is often done using feature processing strategies that can be bolstered by a degree of careful strategic control (Megreya & Burton, 2006).

Also noteworthy is that a measure of visual object recognition (VOSP Silhouettes) correlated with the measures of familiar face recognition (BTWF) and face learning (CFMT), suggesting that it may be worth casting the net more widely to search for a generic visual recognition ability.

Taken together, then, these findings are consistent with the existence of a general face-perception factor. The three main face tasks studied, GFMT, CFMT, and BTWF, are different both in their superficial presentation and in their demand characteristics. Nevertheless, we find that these tests are consistently associated with each other, but only in rather specific case-by-case ways with non-face tests. This lends support to the idea of a general ability with face perception (f) that underlies performance with a wide range of face tasks.

Despite this finding, it is important to emphasise the degree of influence of a putative general face factor. Our observations, and those of Verhallen et al. (2017) suggest that such a factor can account for a maximum of around 25% of the variance across the face tasks studied. That clearly leaves us with much more to understand. In future research it will be important to establish which aspects of face perception have inherently divergent processes, and which are the result of an individual perceiver’s unique history. Studies with much larger cohorts than we have managed to achieve here are becoming possible, largely through on-line testing. It is, therefore, becoming tractable to use an individual-differences approach to study more subtle patterns of association between face-perception tasks and their subcomponents. Such an approach seems very promising in the attempt to understand the relationship between face processing and other human characteristics.

## Conclusions

Understanding the nature of individual differences in ability to perceive and recognise face identity is of importance in real-life contexts ranging from eye-witnessing to passport control. We investigated the relationships between individual differences in the performance of tasks that assessed different aspects of face-identity processing, and the relationships between these tasks and other measures of face perception and broader perceptual, cognitive and personality measures. Our findings were consistent with the existence of a previously hypothesised general face-perception factor suggested by Verhallen et al. (2017), but this was at best able to account for around 25% of the variance in scores. Other influences are also clearly operating, highlighting the potential for different aspects of face-perception abilities to associate with more general tasks in quite specific and differentiated ways.

## Additional file

Additional file 1: Participant-level data for studies 1 and 2. (XLSX 27 kb)

## Abbreviations

BADS: Behavioural Assessment of Dysexecutive Syndrome
BFI: Big Five Personality Inventory
BORB: Birmingham Object Recognition Battery
BTWF: Before They Were Famous task
CFMT: Cambridge Face Memory Test
GFMT: Glasgow Face Matching Test
IRI: Interpersonal Reactivity Index
VOSP: Visual Object and Space Perception Battery
